# Prediction of lymph node status in patients with early-stage cervical cancer based on radiomic features of magnetic resonance imaging (MRI) images

**DOI:** 10.1186/s12880-023-01059-6

**Published:** 2023-08-01

**Authors:** Shuyu Liu, Yu Zhou, Caizhi Wang, Junjie Shen, Yi Zheng

**Affiliations:** 1 Department of Obstetrics and Gynecology, The First Affiliated Hospital of Bengbu Medical College, No.287 Changhuai Road, Longzihu District, Bengbu, Anhui 233004 China; 2 Department of Radiology, The First Affiliated Hospital of Bengbu Medical College, Bengbu, Anhui 233004 China

**Keywords:** Cervical cancer, Lymph node metastasis, Radiomic features, Magnetic resonance imaging, multinomial naive bayes model

## Abstract

**Background:**

Lymph node metastasis is an important factor affecting the treatment and prognosis of patients with cervical cancer. However, the comparison of different algorithms and features to predict lymph node metastasis is not well understood. This study aimed to construct a non-invasive model for predicting lymph node metastasis in patients with cervical cancer based on clinical features combined with the radiomic features of magnetic resonance imaging (MRI) images.

**Methods:**

A total of 180 cervical cancer patients were divided into the training set (n = 126) and testing set (n = 54). In this cross-sectional study, radiomic features of MRI images and clinical features of patients were collected. The least absolute shrinkage and selection operator (LASSO) regression was used to filter the features. Seven machine learning methods, including eXtreme Gradient Boosting (XGBoost), Logistic Regression, Multinomial Naive Bayes (MNB), Support Vector Machine (SVM), Decision Tree, Random Forest, and Gradient Boosting Decision Tree (GBDT) are used to build the models. Receiver operating characteristics (ROC) curve and area under the curve (AUC), accuracy, sensitivity, and specificity were calculated to assess the performance of the models.

**Results:**

Of these 180 patients, 49 (27.22%) patients had lymph node metastases. Five of the 122 radiomic features and 3 clinical features were used to build predictive models. Compared with other models, the MNB model was the most robust, with its AUC, specificity, and accuracy on the testing set of 0.745 (95%CI: 0.740–0.750), 0.900 (95%CI: 0.807–0.993), and 0.778 (95%CI: 0.667–0.889), respectively. Furthermore, the AUCs of the MNB models with clinical features only, radiomic features only, and combined features were 0.698 (95%CI: 0.692–0.704), 0.632 (95%CI: 0.627–0.637), and 0.745 (95%CI: 0.740–0.750), respectively.

**Conclusion:**

The MNB model, which combines the radiomic features of MRI images with the clinical features of the patient, can be used as a non-invasive tool for the preoperative assessment of lymph node metastasis.

**Supplementary Information:**

The online version contains supplementary material available at 10.1186/s12880-023-01059-6.

## Background

Cervical cancer is one of the most common cancers among women, with an estimated 604,127 new cases and 341,831 deaths from cervical cancer worldwide in 2020 [1]. Treatment options for cervical cancer patients with cervical cancer vary depending on the International Federation of Gynecology and Obstetrics (FIGO) stages and the status of lymph nodes [2]. Lymph node metastasis is one of the most important prognostic factors in patients with cervical cancer [3, 4]. The 5-year survival rate for patients with early-stage cervical cancer without lymph node metastases is reported to be 85-90%, but only 50-55% for those with lymph node metastasis [5]. Furthermore, many patients with early-stage cervical cancer may undergo unnecessary lymph node dissection because of undiagnosed or inaccurate assessment of lymph node status [6, 7]. Therefore, accurate assessment of the preoperative lymph node status has an important impact on the treatment and prognosis of patients with cervical cancer.

The criteria for lymph node diagnosis are histopathologic examination after surgical lymphadenectomy or lymph node biopsy [8]. However, these invasive detection methods have some limitations, such as the potential for infection, nerve or vascular injury, and lower extremity lymphedema from the surgical procedure, while biopsy results are influenced by the presence of abnormal lymphatic drainage, the quality of the preoperative lymphadenectomy, and the experience of the surgeons [9–11]. Recently, non-invasive radiomic analysis has been widely used for prognostic assessment [12, 13] and prediction of lymph node metastasis in patients with cervical cancer [14–16].

Previous studies have reported the prediction of lymph node metastasis in patients with cervical cancer based on the radiomic features of different images [14–22]. Liu et al. reported a radiomic features model based on computed tomography (CT) images to predict lymph node metastasis, and the area under the receiver operating characteristic curve (AUC) of their model was 0.859 [20]. Song et al. predicted lymph node metastasis using a radiomic features model of magnetic resonance imaging (MRI) images with an AUC of 0.75 [15]. The radiomic features models for predicting lymph node metastasis in patients with cervical cancer from previous studies are summarized in Table 1. However, most previous studies have explored the predictive effect of only one modeling approach. In addition, the predictive effect of combining radiomic feature with clinical feature models is not well understood. The modeling method and the features used for modeling are important factors that affect the predictive performance of the model.

**Table 1:** Overview of studies using radiological features to predict lymph node metastasis in patients with cervical cancer

Imaging modality	Author, year	Training/testing/validation set	Radiomics/clinical features used	Algorithm	AUC (95%CI)
PET/CT	Zhang, 2022 [18]	104/44/0	14/0	Machine learning	0.786 (0.636–0.895)
MRI	Song, 2021 [15]	90/42/0	7/4	Logistic regression	0.75 (-)
CT	Liu, 2021 [20]	148/74/51	464/0	Artificial neural network	0.859 (0.776–0.941)
MRI	Xiao, 2020 [19]	155/78/0	23/0	Logistic regression	0.883 (0.809–0.957)
CT	Dong, 2020 [21]	176/50/0	5/2	Logistic regression, support vector machine, deep neural network	0.99 (-)
Ultrasound	Jin, 2020 [14]	100/72/0	6/0	Logistic regression	0.77 (0.65–0.88)
CT	Chen, 2020 [22]	104/46/0	2/1	Ridge logistics regression	0.75 (0.53–0.93)
MRI	Kan, 2019 [17]	100/43/0	10/0	Support vector machine	0.754 (0.584–0.924)
MRI	Wu, 2019 [16]	126/63/0	14/1	Support vector machine	0.847 (-)

Note: PET/CT, positron emission tomography/computed tomography; MRI, magnetic resonance imaging; CT, computed tomography; AUC, the area under the receiver operating characteristic curve

Herein, we aimed to establish a model that combines the radiomic features of MRI images with the clinical features of patients to predict lymph node metastasis in patients with cervical cancer. Seven machine learning methods were used to construct models to identify the optimal model.

## Methods

### Study population

Data on cervical cancer patients were obtained from The First Affiliated Hospital of Bengbu Medical College between 2018 and 2021. The identification of the patient’s lymph node metastases was based on histopathological examination. Inclusion criteria were as follows: (1) patients aged ≥ 18 years; (2) patients with primary cervical cancer confirmed by histopathological examination; (3) patients who underwent radical hysterectomy and pelvic lymph node dissection; (4) patients who underwent MRI examination within 2 weeks before hysterectomy; and (5) available clinical information. Exclusion criteria were as follows: (1) patients with combined other malignancies; (2) patients with palliative tumor resection; (3) pregnant or lactating women; (4) patients with preoperative chemotherapy or radiation; (5) patients who underwent biopsy puncture or conization before MRI examination; and (6) patients whose MRI imaging did not meet the requirements for post-processing. This cross-sectional study was approved by the Institutional Review Board of The First Affiliated Hospital of Bengbu Medical College (approval number: 2022KY039), and informed consent was obtained from the patients. All methods were carried out in accordance with relevant guidelines and regulations (declaration of Helsinki).

### MRI image acquisition

All patients underwent pelvic MRI in the supine position using the same 3.0-T MRI scanner (Siemens AG, Munich, Germany) with 8-channel phased-array coil. Before an examination, patients fasted for at least 4 h and filled the bladder moderately. The scanning sequences included axial and sagittal T1-weighted imaging (T1WI) and axial T2-weighted imaging (T2WI). Pelvic T1WI and T2WI images were obtained after injection of 0.2ml/kg gadopentetate dimeglumine for 80–120 s. All images were Digital Imaging and Communications in Medicine (DICOM) format data. The ITK-SNAP (www.itksnap.org) was used to perform manual 3D segmentation of MRI images. Radiologists experienced (more than 10 years) in pelvic MRI diagnosis performed manual segmentation of the lymph node and tumor region of interest (ROI) on each cross-section to validate the segmentation results for each image. The radiologists were blinded to the patient’s lymph node status.

### Radiomics feature extraction

All images are normalized before feature extraction, and the resolution of all images is unified to 1 × 1mm^2^ by interpolation. The interpolation process uses sitkNearestNeighbor as a resampling interpolator to resample the mask to preserve the label values. Radiomic features were extracted from the processed MRI images by Python 3.8 software (PyRadiomics package) [23]. Specifically, the PyRadiomics package’s “RadiomicsFeatureExtractor()” function was used to preprocess the image and create a feature extraction generator, set an optional custom image type using the “enableImageTypes()” function within the generator, and then use the “execute()” function within the generator to calculate the image label of the original image combined with the ROI to obtain the corresponding type of radiomic features. A total of 122 radiomic features were extracted, including First Order Statistics (18 features), Shape-based (2D) (14 features), Gray Level Cooccurrence Matrix (24 features), Gray Level Run Length Matrix (16 features), Gray Level Size Zone Matrix (16 features), Neighbouring Gray Tone Difference Matrix (5 features), Gray Level Dependence Matrix (14 features), and basic image features (5 features).

### Feature selection

The datasets were randomly assigned to the training set and testing set in a ratio of 7:3. All clinical and radiomic features were screened by the least absolute shrinkage and selection operator (LASSO) regression to select the optimal predictive features. Five-fold cross-validation was applied to tune the parameters of the elastic net to select the key features from the high-dimensional feature space and to avoid over-fitting. Finally, 3 clinical features (eosinophil count, red blood cell volume distribution width, squamous cell carcinoma antigen), 2 radiomic features of T1 images (original_firstorder_Range, original_ngtdm_Complexity), and 3 radiomic features of T2 images (diagnostics_Mask-original_VolumeNum, original_glcm_InverseVariance, original_glszm_SmallAreaHighGrayLevelEmphasis) were incorporated into the model.

### Construction, validation, and performance of machine learning model

**Fig. 1:**
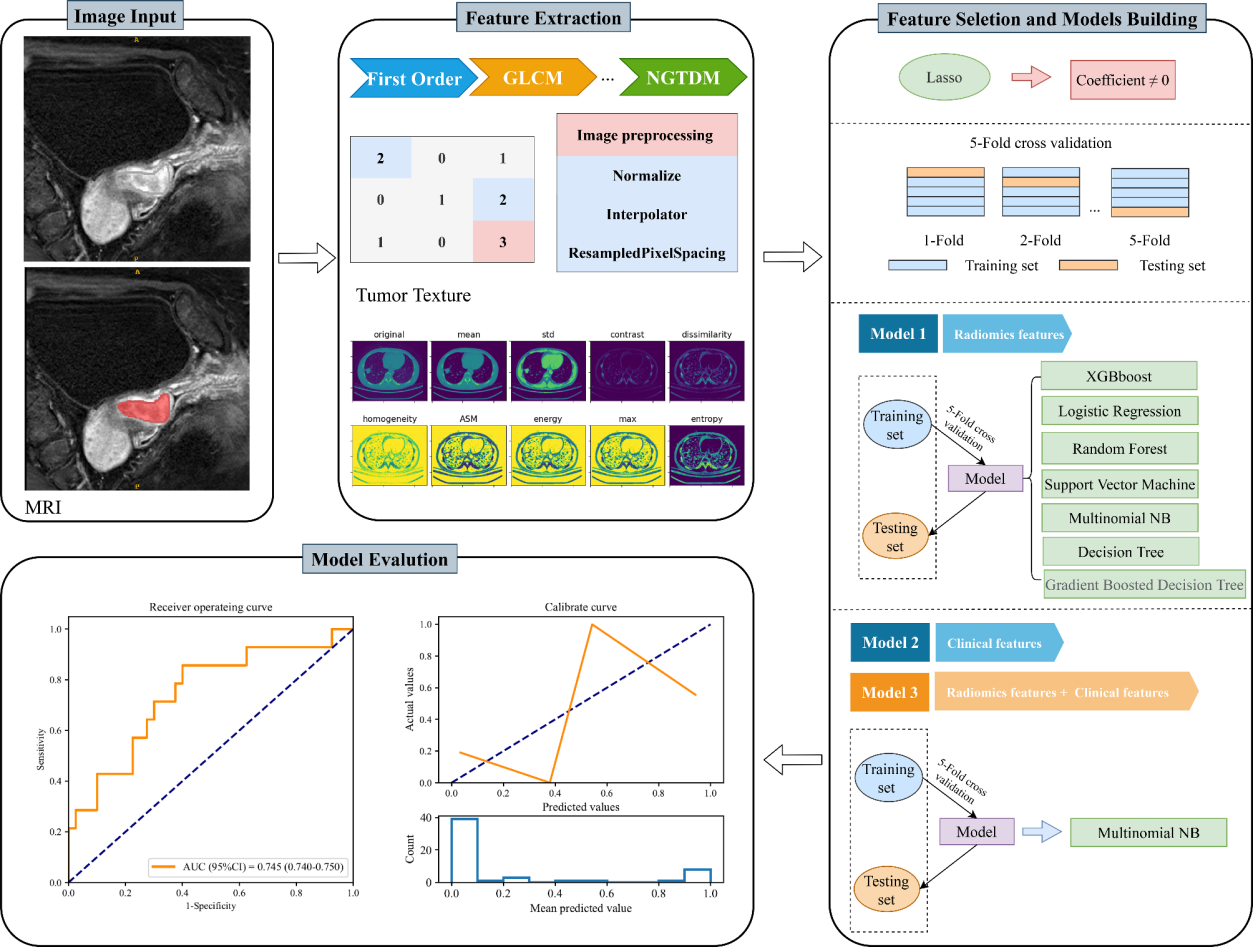
Flowchart of the study. The composition of the prediction system including image input, feature extraction, feature selection, model building, and model evaluation

Seven machine learning methods, including eXtreme Gradient Boosting (XGBoost), Logistic Regression, Multinomial Naive Bayes (MNB), Support Vector Machine (SVM), Decision Tree, Random Forest, and Gradient Boosting Decision Tree (GBDT) are used to build prediction models. All models used 8 clinical and radiomic features that were screened out. Receiver operating characteristics (ROC) curve and area under the curve (AUC), accuracy, sensitivity, specificity, positive predictive value (PPV), and negative predictive value (NPV) were calculated to assess the performance of the models. The ablation analysis was performed to validate the resilience of the model [24, 25]. The model with the best combined performance was selected, and the performance of models with clinical features only, radiomic features only, and combined features was further compared. The flowchart of this study is shown in Fig. 1.

### Statistical analysis

For continuous clinical variables, there were expressed as mean and standard deviation (SD) or median and interquartile range [M (Q1, Q3)], and compared using the Student’s t-test or rank-sum test. Categorical clinical variables were expressed as numbers and percentages [n (%)] and compared using the Chi-square test or Fisher’s exact test. Statistical analyses of clinical data were performed using SAS 9.4 software (SAS Institute Inc., Cary, NC, USA). The extraction of radiomic features and the construction of the model were performed using Python 3.8 software (Python Software Foundation, Delaware, USA). A two-sided P < 0.05 was considered statistically significant.

## Results

### Clinical characteristics of patients

A total of 259 adult women diagnosed with cervical cancer were selected. Of these patients, 79 women were excluded including 73 patients without MRI image data and 6 patients whose MRI images did not meet post-processing requirements. Table 2 shows the characteristics of the 180 included patients. The mean age was 53.07 ± 9.79 years and 97 (53.89%) patients were postmenopausal women. The number of patients in the FIGO stages was 11 (6.11%) for IA2, 2 (1.11%) for IB1, 45 (25.00%) for IB2, 53 (29.44%) for IB3, 18 (10.00%) for IIA1, and 51 (28.33%) for IIA2. The median squamous cell carcinoma antigen (SCC-Ag) level was 1.63 (0.84, 4.55) ng/mL. There were 49 (27.22%) patients with lymph node metastasis and 131 (72.78%) patients without lymph node metastasis.

**Table 2:** Clinical characteristics of patients

Variables	Total (n = 180)	Non-LNM (n = 131)	LNM (n = 49)	*P*
Age, years, Mean ± SD	53.07 ± 9.79	52.87 ± 10.20	53.61 ± 8.69	0.652
Marital status, n (%)				1.000
Unmarried	2 (1.11)	2 (1.53)	0 (0.00)	
Married	178 (98.89)	129 (98.47)	49 (100.00)	
Reproductive history, n (%)				0.576
No	4 (2.22)	4 (3.05)	0 (0.00)	
Yes	176 (97.78)	127 (96.95)	49 (100.00)	
Weight, kg, Mean ± SD	62.13 ± 9.88	61.61 ± 10.11	63.54 ± 9.21	0.244
Menopausal status, n (%)				0.630
Non-menopausal	67 (37.22)	50 (38.17)	17 (34.69)	
Peri-menopause	16 (8.89)	10 (7.63)	6 (12.24)	
Post-menopausal	97 (53.89)	71 (54.20)	26 (53.06)	
FIGO stage, n (%)				0.018
IA2	11 (6.11)	4 (3.05)	7 (14.29)	
IB1	2 (1.11)	2 (1.53)	0 (0.00)	
IB2	45 (25.00)	38 (29.01)	7 (14.29)	
IB3	53 (29.44)	39 (29.77)	14 (28.57)	
IIA1	18 (10.00)	10 (7.63)	8 (16.33)	
IIA2	51 (28.33)	38 (29.01)	13 (26.53)	
White blood cells, 10^9/L, M (Q_1_, Q_3_)	6.85 (5.64,8.87)	6.92 (5.65,8.72)	6.47 (5.62,9.24)	0.684
Eosinophil count, 10^9/L, M (Q_1_, Q_3_)	0.10 (0.05,0.20)	0.11 (0.05,0.21)	0.08 (0.06,0.13)	0.251
Basophil count, 10^9/L, M (Q_1_, Q_3_)	0.01 (0.00,0.02)	0.01 (0.00,0.02)	0.01 (0.00,0.02)	0.627
Monocyte count, 10^9/L, M (Q_1_, Q_3_)	0.43 (0.34,0.54)	0.43 (0.34,0.54)	0.41 (0.33,0.51)	0.483
Red blood cells, 10^12/L, Mean ± SD	4.14 ± 0.58	4.16 ± 0.57	4.10 ± 0.61	0.574
Hemoglobin, g/L, Mean ± SD	120.68 ± 18.14	121.92 ± 17.45	117.35 ± 19.68	0.132
Hematocrit, %, Mean ± SD	36.23 ± 4.92	36.53 ± 4.71	35.44 ± 5.42	0.187
Mean corpuscular volume, fL, Mean ± SD	87.48 ± 6.47	87.86 ± 6.52	86.45 ± 6.29	0.195
Mean corpuscular hemoglobin, pg, Mean ± SD	29.16 ± 2.79	29.35 ± 2.76	28.66 ± 2.82	0.138
Mean erythrocyte hemoglobin concentration, g/L, Mean ± SD	332.57 ± 13.59	333.53 ± 13.10	330.00 ± 14.64	0.122
Red blood cell distribution width- coefficient of variation, %, Mean ± SD	13.57 ± 1.91	13.41 ± 1.69	14.01 ± 2.37	0.110
Red blood cell distribution width-standard deviation, %, Mean ± SD	42.75 ± 3.66	42.53 ± 3.37	43.34 ± 4.33	0.240
Mean platelet volume, fL, Mean ± SD	10.76 ± 1.13	10.80 ± 1.11	10.66 ± 1.18	0.479
Thrombocytocrit, %, Mean ± SD	0.28 ± 0.79	0.28 ± 0.81	0.29 ± 0.74	0.455
Platelet distribution width, %, Mean ± SD	13.09 ± 2.54	13.15 ± 2.69	12.92 ± 2.11	0.587
Platelet large cell ratio, %, Mean ± SD	31.13 ± 9.27	31.21 ± 9.58	30.92 ± 8.50	0.853
Squamous cell carcinoma antigen, ng/mL, M (Q_1_, Q_3_)	1.63 (0.84,4.55)	1.50 (0.80,3.80)	3.60 (1.20,10.76)	< 0.001

Note: LNM, lymph node metastasis; FIGO, the International Federation of Gynecology and Obstetrics

### Prediction performance of models

**Fig. 2:**
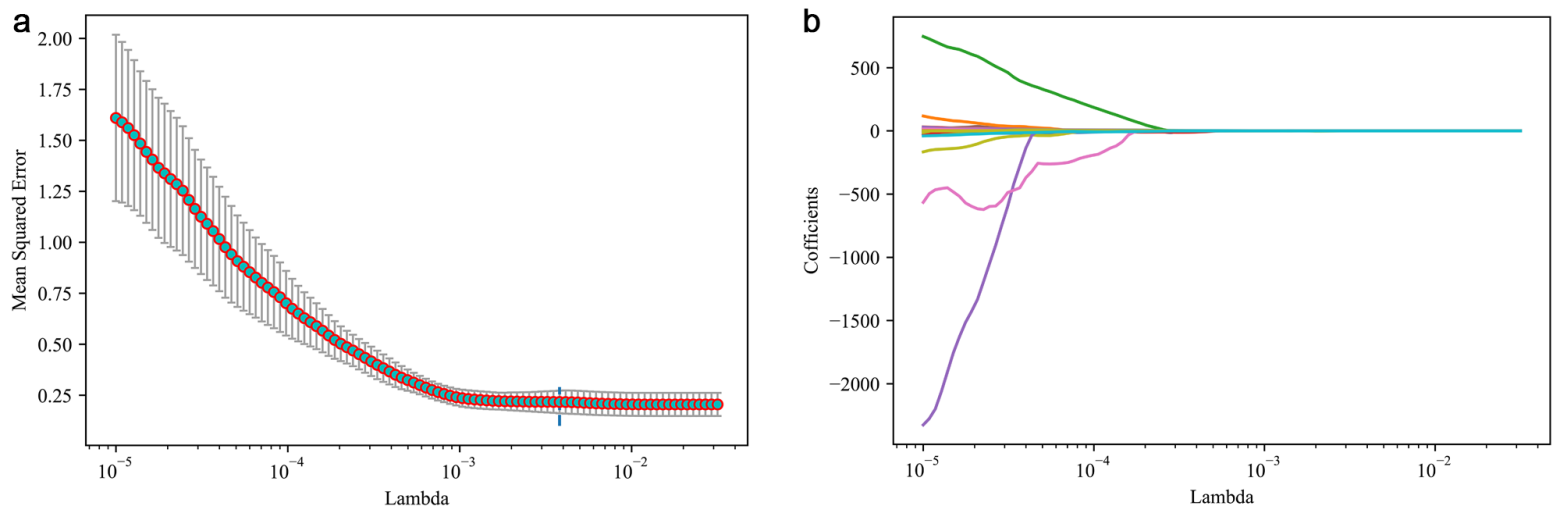
Feature selection using the least absolute shrinkage and selection operator (LASSO) regression. **(a)** changes in mean squared error during LASSO regression screening; **(b)** Changes in the coefficient profiles during LASSO regression screening

**Fig. 3:**
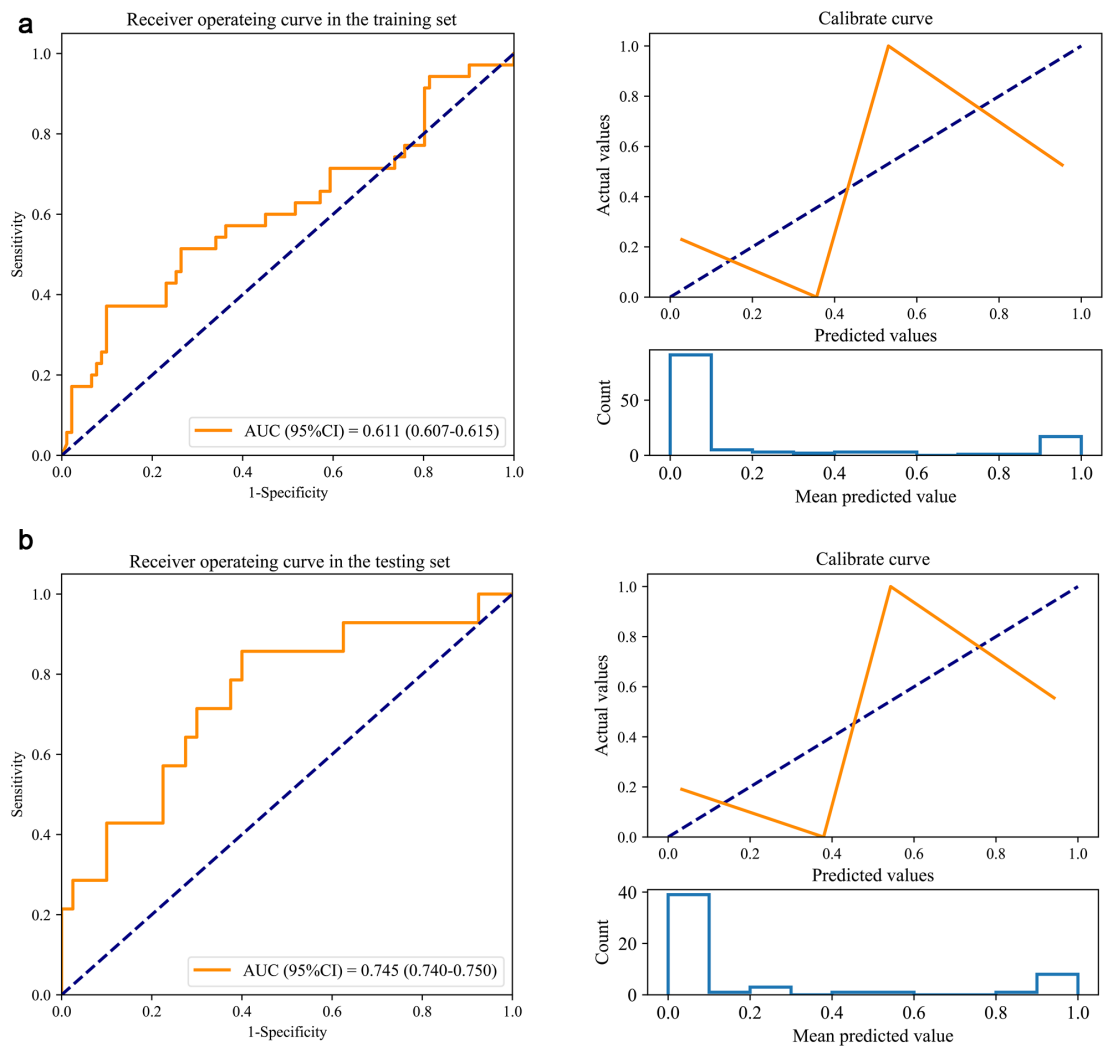
The receiver operator characteristic (ROC) curves and calibrate curve of the Multinomial Naive Bayes (MNB) model in the training set and testing set. **(a)** ROC curves and calibrate curve in the training set; **(b)** ROC curves and calibrate curve in the testing set

Three clinical features and five radiomic features were selected to build the prediction model. The process of screening features by LASSO regression is presented in Fig. 2. Table 3 shows the performance of different models in predicting lymph node metastasis in patients with cervical cancer. In the training set, the AUCs were 0.939 (95%CI: 0.938–0.940) for the XGBoost model, 0.687 (95%CI: 0.683–0.690) for the Logistic Regression model, 0.611 (95%CI: 0.607–0.615) for the MNB model, 0.830 (95%CI: 0.827–0.832) for the SVM model, 0.691 (95%CI: 0.688–0.694) for the Decision Tree model, 0.875 (95%CI: 0.873–0.877) for the Random Forest model, and 0.997 (95%CI: 0.997–0.997) for the GBDT model. In the testing set, the performance parameters of the models, except for the MNB model, differed significantly between the training and testing sets. The AUC, specificity, and accuracy of the MNB model in the testing set were 0.745 (95%CI: 0.740–0.750), 0.900 (95%CI: 0.807–0.993), and 0.778 (95%CI: 0.667–0.889), respectively. Therefore, the MNB model was used to predict preoperative lymph node metastasis in patients with cervical cancer. The ROC curves and calibrate curves of the MNB model in the training set and testing set are shown in Fig. 3. In addition, the ablation analysis was performed based on the parameter “alpha” of the MNB model. The results showed that when the parameter “alpha” of the MNB model was 5, the test accuracy was best at 77.78% and the test loss was the lowest at 0.8032% (Supplement Table 1).

**Table 3:** Performances of different models in predicting lymph node metastasis in patients with cervical cancer

Models	Cutoff	Sensitivity (95%CI)	Specificity (95%CI)	PPV (95%CI)	NPV (95%CI)	AUC (95%CI)	Accuracy (95%CI)
XGBoost model							
Training set	0.413	0.886(0.780–0.991)	0.890(0.826–0.954)	0.756(0.625–0.888)	0.953(0.908–0.998)	0.939(0.938–0.940)	0.889(0.834–0.944)
Testing set	0.413	0.786(0.571-1.000)	0.600(0.448–0.752)	0.407(0.222–0.593)	0.889(0.770-1.000)	0.721(0.716–0.727)	0.648(0.521–0.776)
**Logistic Regression model**							
Training set	0.263	0.657(0.500-0.814)	0.670(0.574–0.767)	0.434(0.301–0.567)	0.836(0.751–0.921)	0.687(0.683–0.690)	0.667(0.584–0.749)
Testing set	0.263	1.000(1.000–1.000)	0.150(0.039–0.261)	0.292(0.163–0.420)	1.000(1.000–1.000)	0.812(0.809–0.816)	0.370(0.242–0.499)
**MNB model**							
Training set	0.508	0.371(0.211–0.532)	0.901(0.840–0.962)	0.591(0.385–0.796)	0.788(0.710–0.867)	0.611(0.607–0.615)	0.754(0.679–0.829)
Testing set	0.508	0.429(0.169–0.688)	0.900(0.807–0.993)	0.600(0.296–0.904)	0.818(0.704–0.932)	0.745(0.740–0.750)	0.778(0.667–0.889)
**SVM model**							
Training set	0.094	0.829(0.704–0.953)	0.824(0.746–0.902)	0.644(0.505–0.784)	0.926(0.869–0.983)	0.830(0.827–0.832)	0.825(0.759–0.892)
Testing set	0.094	0.929(0.794-1.000)	0.100(0.007–0.193)	0.265(0.142–0.389)	0.800(0.449-1.000)	0.696(0.690–0.703)	0.315(0.191–0.439)
**Decision Tree model**							
Training set	0.668	0.886(0.780–0.991)	0.440(0.338–0.542)	0.378(0.273–0.483)	0.909(0.824–0.994)	0.691(0.688–0.694)	0.563(0.477–0.650)
Testing set	0.668	0.643(0.392–0.894)	0.775(0.646–0.904)	0.500(0.269–0.731)	0.861(0.748–0.974)	0.724(0.719–0.729)	0.741(0.624–0.858)
**Random Forest model**							
Training set	0.251	0.829(0.704–0.953)	0.758(0.670–0.846)	0.569(0.433–0.705)	0.920(0.859–0.981)	0.875(0.873–0.877)	0.778(0.705–0.850)
Testing set	0.251	0.643(0.392–0.894)	0.650(0.502–0.798)	0.391(0.192–0.591)	0.839(0.709–0.968)	0.684(0.678–0.690)	0.648(0.521–0.776)
**GBDT model**							
Training set	0.273	0.971(0.916-1.000)	0.978(0.948-1.000)	0.944(0.870-1.000)	0.989(0.967-1.000)	0.997(0.997–0.997)	0.976(0.950-1.000)
Testing set	0.273	0.429(0.169–0.688)	0.750(0.616–0.884)	0.375(0.138–0.612)	0.789(0.660–0.919)	0.651(0.645–0.657)	0.667(0.541–0.792)

Note: PPV, positive predictive value; NPV, negative predictive value; AUC, receiver operating characteristics curve area under the curve; XGBoost, eXtreme Gradient Boosting; MNB, Multinomial Naive Bayes; SVM, Support Vector Machine; GBDT, Gradient Boosting Decision Tree

### Performance comparison of models using different features

A comparison of the prediction performance of the MNB model using different features is shown in Table 4. In the testing set, the AUCs of the MNB model with clinical features only, radiomic features only, and combined features were 0.698 (95%CI: 0.692–0.704), 0.632 (95%CI: 0.627–0.637), and 0.745 (95%CI: 0.740–0.750), respectively. Compared with other feature models, the MNB model with radiomic features combined with clinical features had a better performance for predicting preoperative lymph node metastasis in patients with cervical cancer.

**Table 4:** Comparison of the prediction performance of the Multinomial Naive Bayes (MNB) model using different features

Models	Cutoff	Sensitivity (95%CI)	Specificity (95%CI)	PPV (95%CI)	NPV (95%CI)	AUC (95%CI)	Accuracy (95%CI)
Radiomic features + clinical features model							
Training set	0.508	0.371(0.211–0.532)	0.901(0.840–0.962)	0.591(0.385–0.796)	0.788(0.710–0.867)	0.611(0.607–0.615)	0.754(0.679–0.829)
Testing set	0.508	0.429(0.169–0.688)	0.900(0.807–0.993)	0.600(0.296–0.904)	0.818(0.704–0.932)	0.745(0.740–0.750)	0.778(0.667–0.889)
Clinical features model							
Training set	0.116	0.514(0.349–0.680)	0.736(0.646–0.827)	0.429(0.279–0.578)	0.798(0.712–0.884)	0.641(0.637–0.644)	0.675(0.593–0.756)
Testing set	0.116	0.500(0.238–0.762)	0.800(0.676–0.924)	0.467(0.214–0.719)	0.821(0.700-0.941)	0.698(0.692–0.704)	0.722(0.603–0.842)
Radiomic features model							
Training set	0.200	0.657(0.500-0.814)	0.440(0.338–0.542)	0.311(0.205–0.416)	0.769(0.655–0.884)	0.523(0.520–0.527)	0.500(0.413–0.587)
Testing set	0.200	0.929(0.794-1.000)	0.350(0.202–0.498)	0.333(0.185–0.481)	0.933(0.807-1.000)	0.632(0.627–0.637)	0.500(0.367–0.633)

Note: PPV, positive predictive value; NPV, negative predictive value; AUC, receiver operating characteristics curve area under the curve

## Discussion

In this study, we used the radiomic features of MRI images combined with clinical features of patients to predict lymph node metastasis in patients with cervical cancer. We compared the prediction performance of seven machine learning models, among which the MNB model had the best prediction effects in the testing set with an AUC of 0.745. Furthermore, the MNB model with a combination of radiomic and clinical features had better prediction effects than the model with single radiomic features and clinical features.

Predictive models based on radiomic features have been widely used to predict the prognosis of many diseases [26–28]. The radiomic feature model provides a non-invasive evaluation method for patient prognosis assessment [29]. Compared to traditional invasive evaluation methods such as biopsy, the radiomic feature models can reduce patient harm and avoid excessive biopsies on patients [30]. Many factors affect the prediction effectiveness of the radiomic feature model, such as the type of image (ultrasound images, CT images, MRI images), the ROI classification, and the method of model construction [31]. Jin et al. constructed a logistic regression model for predicting lymph node metastasis in patients with cervical cancer using the radiomics features of ultrasound images, and the AUC of the model was 0.77 [14]. Chen et al. used clinical features combined with radiomic features of CT images to establish a ridge logistics regression model for predicting lymph node metastasis, and the AUC of the model was 0.75 [22]. More studies have used the radiomic features of MRI images to construct predictive models for lymph node metastasis in patients with cervical cancer [15, 17, 32]. MRI is useful for detecting lymph node metastasis, especially when the tumor size is greater than 4 cm (accuracy 84%) [33]. However, MRI images may miss normal-sized lymph node metastasis and cannot reliably distinguish inflammatory lymph node enlargement from cancer-infiltrating lymph nodes. The radiomic analysis may be able to compensate for this limitation of images alone. This study used the radiomic features of MRI images combined with clinical features to develop a model for predicting lymph node metastasis in patients with cervical cancer. In contrast to previous studies, the current study compared the predictive effects of seven different models. In radiomic analysis, the predictive performance of different machine learning models is different.

Our study screened 3 clinical features and 5 radiomic features for modeling. In clinical features, eosinophils have been reported to be associated with lymph node metastasis in patients with tumors [34]. Red blood cell volume distribution width reflects the size variability of circulating erythrocytes related to chronic inflammation, which is an important influencing factor in the progression of various cancer diseases [35]. Preoperative serum squamous carcinoma antigen levels have also been found to be potentially useful predictors of early lymph node metastasis in squamous cervical cancer [36]. Among our radiomic features, 2 were from T1WI images and 3 were from T2WI images. T2WI can provide information on tumor morphology and stroma information, and T1W can reflect tumor microenvironment and aggressiveness by showing microvascular density and perfusion [37]. The original_firstorder_Range feature is the range of gray values in the ROI, reflecting the heterogeneity within the tumor. The original_ngtdm_Complexity, original_glcm_InverseVariance, and original_glszm_SmallAreaHighGrayLevelEmphasis features are all texture features of the image, and texture can quantify information that is difficult to be perceived simply by vision, such as texture patterns or tissue distribution within the tumor [38]. The original_ngtdm_Complexity indicates the complexity of the image, i.e., the image is non-uniform and there are many rapid changes in gray level intensity. The original_glcm_InverseVariance feature is a measure of image homogeneity. The original_glszm_SmallAreaHighGrayLevelEmphasis feature represents the proportion of the joint distribution of small-sized areas with high gray values in the image, which reflects the gray level changes within peritumoral regions. These radiomic features reflect changes in the tumor and peritumor area.

Among these models, both the XGBoost model and the GBDT model showed good prediction performance in the training set, and their AUCs were more than 0.9. However, these models including the XGBoost model, the GBDT model, the SVM model, the Logistic Regression model, and the Random forest model had significant differences in performance on the training set and testing set. This may suggest an overfitting of these models. The prediction performance of the MNB model was robust in both the training set and testing set, and the AUC in the testing set was 0.745. Furthermore, we compared the predictive performance of the models using radiomic features, clinical features, and radiomic features combined with clinical features, respectively. The results demonstrated that the model with radiomic features combined with clinical features had a better prediction effect. Previous studies have shown that radiomic features combined with patient or tumor characteristics can improve medical decisions through clinical decision support systems, thereby improving diagnostic, prognostic, and predictive accuracies, and facilitating therapeutic research [30, 39]. The prediction performance of the current model has not been significantly improved compared to previous studies. The main direction of future study is to further improve the predictive performance of the model because the AUC of the model was about 0.75 in both the current and previous studies. Furthermore, future studies may need to focus on the metastasis of smaller-diameter lymph nodes.

This study used the radiomic features of MRI images and the clinical features of patients to construct a model for predicting lymph node metastasis in patients with cervical cancer. We compared the prediction performance of seven machine learning models. This study may provide a reference for the selection of different machine learning prediction models. There were several limitations in our study. First, the sample size of patients recruited in this single-center retrospective study was small, and a larger sample size from multiple centers is needed to confirm the predictive effect of our model. Second, serological biomarkers associated with cervical cancer, such as carbohydrate antigen 125 (CA125), carbohydrate antigen 153 (CA153), carbohydrate antigen 199 (CA199) and carcinoembryonic antigen (CEA), were not used to build predictive models due to too much missing data. Third, although the model performed well in internal validation, external validation of the model was also required. Fourth, prospective study design and rigorous study procedures in future studies are needed based on the Radiomics Quality Score of Lambin et al. [39].

## Conclusions

This study used the radiomic features of MRI images combined with the clinical features of patients to predict lymph node metastasis in patients with cervical cancer. Seven machine learning methods were used to build models to identify the best modeling method. The MNB model showed the most robust predictive performance, which might be used as a non-invasive tool for the preoperative assessment of lymph node metastasis. Future studies may need to further improve the predictive performance of the model.

## Electronic supplementary material

Below is the link to the electronic supplementary material.

Supplementary Material 1

## Data Availability

The datasets used and/or analyzed during the current study are available from the corresponding author on reasonable request.

## List of abbreviations

FIGO: International Federation of Gynecology and Obstetrics
DICOM: Digital Imaging and Communications in Medicine
ROI: Region of interest
LASSO: Least absolute shrinkage and selection operator
MNB: Multinomial Naive Bayes
SVM: Support Vector Machine
GBDT: Gradient Boosting Decision Tree
ROC: Receiver operating characteristics
AUC: Area under the curve
PPV: Positive predictive value
NPV: Negative predictive value
