# Supplementary material for: Prediction of lymph node status in patients with early-stage cervical cancer based on radiomic features of magnetic resonance imaging (MRI) images
Source: BMC Med Imaging. 2023 Aug 1;23:101. doi: 10.1186/s12880-023-01059-6 (PMC10392004; doi:10.1186/s12880-023-01059-6)
Supplement: Supplementary file 1 — Supplementary Material 1 [file 12880_2023_1059_MOESM1_ESM.docx]

**Supplement Table 1**. The ablation analysis of the Multinomial Naive Bayes (MNB) model.

| **Case Study** | **alpha** | **Test Accuracy (%)** | **Test Loss (%)** | **Performance** |
| --- | --- | --- | --- | --- |
| **1** | **5** | **77.78** | **0.8032** | **Identical** |
|  | 7 | 77.78 | 0.8057 | Loss_Increased |
|  | 9 | 77.78 | 0.8091 | Loss_Increased |
|  | 11 | 75.93 | 0.8133 | Loss_Increased |
|  | 13 | 75.93 | 0.8180 | Loss_Increased |
|  | 15 | 75.93 | 0.8234 | Loss_Increased |
